# Helium, inorganic and organic carbon isotopes of fluids and gases across the Costa Rica convergent margin

**DOI:** 10.1038/s41597-019-0302-4

**Published:** 2019-11-25

**Authors:** Peter H. Barry, Mayuko Nakagawa, Donato Giovannelli, J. Maarten de Moor, Matthew Schrenk, Alan M. Seltzer, Elena Manini, Daniele Fattorini, Marta di Carlo, Francesco Regoli, Katherine Fullerton, Karen G. Lloyd

**Affiliations:** 1 Marine Chemistry and Geochemistry Department, Woods Hole Oceanographic Institution, Woods Hole, MA USA; 2 Earth-Life Science Institute, Tokyo Institute for Technology, Tokyo, Japan; 3 Department of Biology, University of Naples “Federico II”, Naples, Italy; 4 Department of Marine and Coastal Science, Rutgers University, New Brunswick, NJ USA; 5 Institute for Marine Biological and Biotechnological Resources, National Research Council of Italy, CNR-ISMAR, Ancona, Italy; 6 Observatorio Volcanológico y Sismológico de Costa Rica (OVSICORI), Universidad Nacional, Heredia, Costa Rica; 7 Department of Earth and Environmental Sciences, Michigan State University, East Lansing, MI USA; 8 Dipartimento di Scienze della Vita e dell’Ambiente (DISVA), Università Politecnica delle Marche (UNIVPM), Ancona, Italy; 9 CoNISMa, Consorzio Nazionale Interuniversitario Scienze del Mare, Rome, Italy; 10 Department of Microbiology, University of Tennessee, Knoxville, TN USA

**Keywords:** Hydrology, Carbon cycle, Solid Earth sciences

## Abstract

In 2017, fluid and gas samples were collected across the Costa Rican Arc. He and Ne isotopes, C isotopes as well as total organic and inorganic carbon concentrations were measured. The samples (n = 24) from 2017 are accompanied by (n = 17) samples collected in 2008, 2010 and 2012. He-isotopes ranged from arc-like (6.8 R_A_) to crustal (0.5 R_A_). Measured dissolved inorganic carbon (DIC) δ^13^C_VPDB_ values varied from 3.55 to −21.57‰, with dissolved organic carbon (DOC) following the trends of DIC. Gas phase CO_2_ only occurs within ~20 km of the arc; δ^13^C_VPDB_ values varied from −0.84 to −5.23‰. Onsite, pH, conductivity, temperature and dissolved oxygen (DO) were measured; pH ranged from 0.9–10.0, conductivity from 200–91,900 μS/cm, temperatures from 23–89 °C and DO from 2–84%. Data were used to develop a model which suggests that ~91 ± 4.0% of carbon released from the slab/mantle beneath the Costa Rican forearc is sequestered within the crust by calcite deposition with an additional 3.3 ± 1.3% incorporated into autotrophic biomass.

## Abstract (summary)

**Table:** 

Measurement(s)	temperature of air • pH • Total Organic Carbon • carbon-13 atom • dissolved inorganic carbon content • dissolved organic carbon content • carbon dioxide • helium-3 atom • helium-4 atom • methane
Technology Type(s)	Temperature Probe Device • pH sensor • mass spectrometer
Factor Type(s)	time • location where data was collected
Sample Characteristic - Environment	volcanic arc • fumarole • hot spring • seep
Sample Characteristic - Location	Costa Rica

Machine-accessible metadata file describing the reported data: 10.6084/m9.figshare.10293350

## Background & Summary

**Fig. 1:**
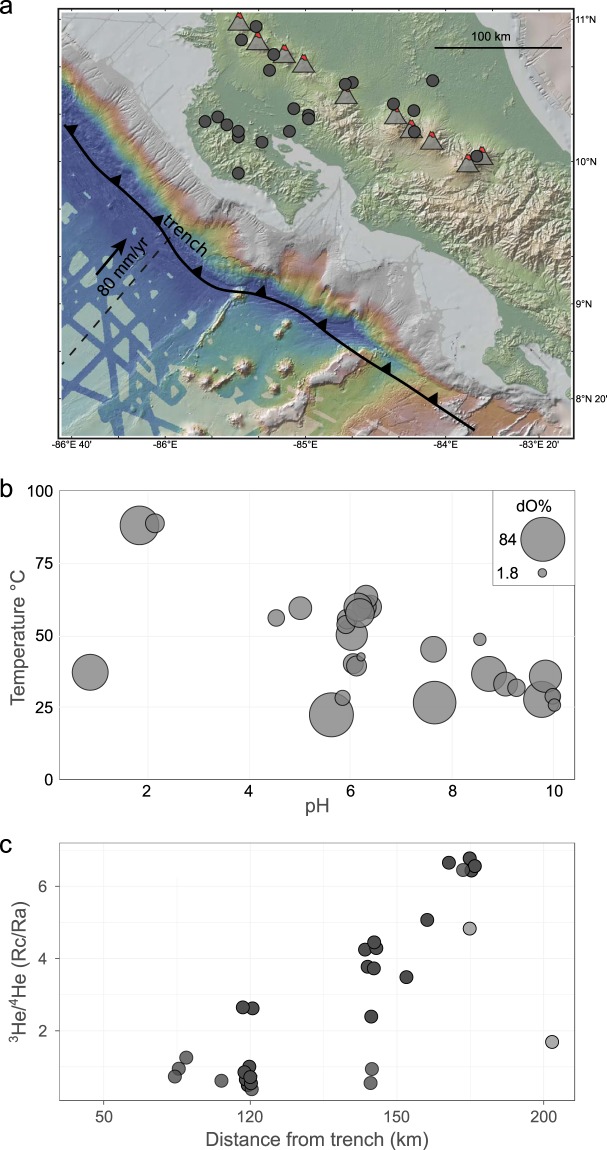
Map, pH, temperature and helium isotopes in Costa Rica. Panel a - map of Costa Rica showing sample localities as circles and volcanic centers as triangles. Panel b - Temperature vs. pH of all sites sampled. The size of the circle corresponds to dissolved oxygen saturation (largest circle = 84% dissolved oxygen saturation, smallest circle = 1.8% dissolved oxygen saturation). Panel c - Helium isotopes vs. distance from trench (i.e., trench as shown in Panel a).

**Fig. 2:**
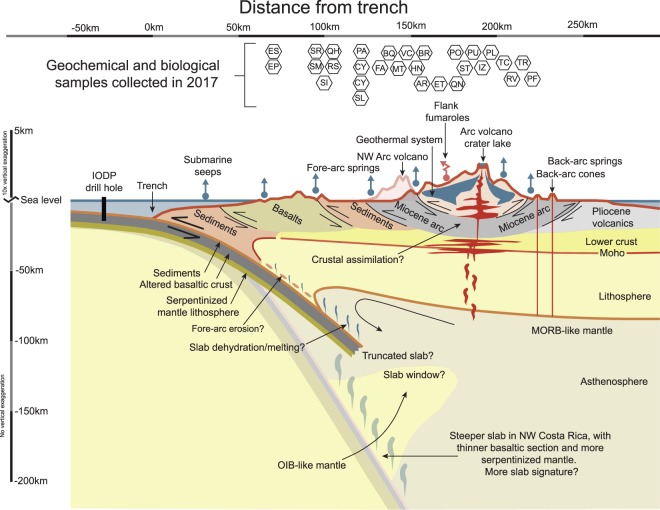
Schematic cross section of a subduction zone. Note that the sample localities (two letter initials) are shown as a function of distance from the trench.

Carbon and other volatiles are transported from Earth’s surface into the mantle at subduction margins (Figs. 1 and 2). The efficiency of this transfer has important implications for the nature and scale of geochemical heterogeneities in Earth’s deep (mantle) and shallow (crustal) reservoirs. However, the proportion of volatiles released outside of the arc (i.e., in the forearc and backarc) are not well-constrained compared to fluxes from the volcanic-front. In a recent study^1^, we used helium and carbon isotope data from deeply-sourced springs along two cross-arc transects to show that carbon is likely sequestered within the crust by calcite deposition and incorporated into biomass through microbial chemolithoautotrophy.

As carbon moves between Earth’s surface (atmosphere and ocean), crust and mantle, it is involved in a number of geological, geochemical, and biological cycles, each of which operates on dramatically different temporal and spatial scales. Helium, on the other hand, acts as a tracer of deep subsurface inputs, since it is not subject to biological or chemical transformations. One of the most important physical processes linking the deep and shallow carbon cycles is subduction, which transports surface-derived carbon into the mantle. During subduction, volatile-rich fluids are released from the downgoing slab (oceanic crust and upper mantle). These fluids are thought to migrate through the overlying mantle-wedge and crust, and are ultimately released across the forearc, volcanic arc-front and backarc. The main goal of the “Biology Meets Subduction” project^1^ was to understand how biogeochemical processes shape the isotope signatures observed in surface manifestations in a subduction setting – namely Costa Rica. Until now, few studies have combined geological and biological observations to determine the dominant processes across a regional scale, despite the fact that much of the forearc subsurface is at low temperatures (<100 °C) that are conducive to microbial life.

Previous carbon budgets for convergent margins typically ignored emissions from the forearc and backarc^2–4^. However, it is not clear whether the lack of obvious high emission sources in these regimes reflects a lack of deep CO_2_ production, or if secondary processes in the upper plate^5–7^ mask diffuse but significant CO_2_ release. In order to determine what the dominant processes (chemical vs. biological) in the forearc were, we sampled two comprehensive transects across the arc and found coherent isotope trends in C and He. Carbon and He isotope compositions increased in the heavier isotope systematically moving from forearc to arc (Fig. 1). The increase in He isotope (Rc/Ra) values is attributed to a more significant mantle input in arc regions, however there is a persistent deep contribution even in the forearc springs, which can clearly be seen in elevated He isotope ratios. The δ^13^C values in both dissolved inorganic carbon (DIC) and dissolved organic carbon (DOC) were lowest in the outer forearc, which we attributed to extensive calcite precipitation in lower temperature environments. Using these data, we quantitatively modeled C sequestration by calcite precipitation and estimated the rate thereof in the forearc. We then compared these estimates to the total volatile budget of the subduction zone. Our estimates indicate that shallow processes (i.e., calcite precipitation) have a significant effect on volatile fluxes in the forearc and thus have important implications for the overall recycling efficiency of carbon, suggesting that between 0.2% and 19% less CO_2_ is transported into the deep mantle than was previously estimated. These comprehensive data provide a framework for understanding an array of biogeochemical processes impacting volatiles in subduction zones, and contribute fundamental information that can be integrated into models relating plate tectonics and climate.

## Methods

Sampled hot springs span two transects across the Costa Rica volcanic arc from a latitude of 9.561575°N to 10.89837°N (Figs. 1 & 2), moving from the coastline (altitude of 36 meters above sea level), to several of the volcano summits (maximum altitude of 2,335 m above sea level). The dataset represents a cohesive snapshot of helium and carbon across the Northern Costa Rica volcanic arc, the forearc (Nicoya Peninsula), central Costa Rica and regions within the Caribbean lowlands. Methods were previously described^1^, however more details about the procedures are provided here.

### Sample collection

Gas, fluids and sediments samples (n = 24) were collected from fumaroles, wells, bubbling springs and naturally flowing fluid springs over the course of two weeks February 2017 (Fig. 1). Within each pool, the main inlet was identified by using a combination of field observation, pH and temperature analyses. The objective was to sample the pristine gases and fluids as they emerged from the subsurface. Sediments were collected in close proximity (within centimeters when available) to the inlet. These samples were complemented by an additional (n = 17) samples collected during previous field campaigns in 2008, 2010 and 2012 from similar types of sites. He and C isotope data from the initial reconnaissance field campaigns were produced in the Fluids and Volatiles Laboratory at Scripps Institute for Oceanography, USA (SIO), whereas data from 2017 are from the Noble Laboratory at the University of Oxford, UK (He-isotopes), National University of Costa Rica (UNA), Costa Rica (C-isotopes), and the Earth-Life Science Institute, ELSI, Tokyo Institute for Technology, Japan (C-isotopes). Hydrocarbons and organic matter data were produced at the Department of Life Sciences of the Polytechnic University of Marche (UNIVM), Italy (hydrocarbons).

### SIO C and He cleanup

**Fig. 3:**
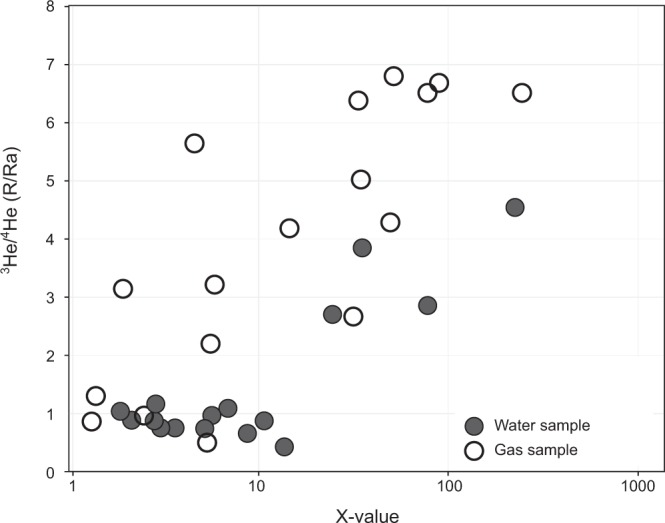
Helium isotopes (^3^He/^4^He) versus X values. The X values are air-normalized ^4^He/^20^Ne; considering solubility in water for fluid samples. The majority of samples have high (>5) X values, indicating minimal air-contributions to samples.

Gas and water samples from the 2008, 2010 and 2012 campaigns were analyzed at SIO for He and C isotopes using instrumentation and protocols described previously^3,8–12^. All samples were extracted on a dedicated ultra-high vacuum (UHV) extraction line^8^, which was used to separate light noble gases (He and Ne) from condensable gases (Fig. 3). Following sample inlet, all fluid samples were acidified with phosphorus pentoxide to ensure complete release of CO_2_ and, as a result, measured CO_2_ abundances corresponded to the total dissolved inorganic carbon (DIC) content. A glass trap held at the temperature of a mixture of acetone and dry ice was used to isolate water vapor and a stainless-steel U-tube, held at liquid nitrogen temperature, was used to separate CO_2_ from non-condensable volatiles. The remaining light noble gases (He and Ne) were then isolated using a hot (700 °C) Ti-getter and charcoal finger held at liquid nitrogen temperature, which effectively removed active gases (N_2_, CO, CH_4_) and heavy noble gases (Ar, Kr and Xe), respectively. A calibrated fraction of the He and Ne gas was captured in an Ar-glass breakseal for transfer to a MAP-215 noble gas mass spectrometer for He isotope analysis. Next, the CO_2_ fraction was transferred to a Pyrex breakseal for transfer to a dedicated CO_2_ cleanup line. The condensable CO_2_ sample fraction was further purified on a separate cleanup and quantification line, constructed from Pyrex glass, whereby CO_2_ was separated from any sulfur-bearing species using a variable temperature trap. Following cleanup, the total amount of CO_2_ was measured using a capacitance manometer in a calibrated volume. Lastly, CO_2_ was re-frozen into a Pyrex tube for transfer to a Thermo Finnigan Delta XP^plus^ isotope ratio mass spectrometer for carbon isotopic (δ^13^C) analysis.

### SIO noble gas isotope analysis

He and Ne analyses were carried out on a MAP-215 noble gas mass spectrometer at SIO^9–11^. First, the gas was released from the breakseal and subjected to further purification by sequential exposure to a 750 °C hot Ti-getter, an activated charcoal trap (held at −196 °C), a Società Apparecchi Electrici e Scientifici (SAES) getter, and a cryogenic trap lined with activated charcoal (held at <20 K). Helium was released from the cryogenic trap by increasing the temperature to 35 K, whereas Ne was released at 90 K. ^3^He/^4^He and ^4^He/^20^Ne ratios were measured in static mode and calibrated against standard aliquots of air run at least twice a day under identical conditions. The blank contribution to the ^4^He signal was less than 3% for all gas and water samples.

### SIO carbon isotope analysis

For isotope analysis, the CO_2_ aliquot was inlet into a Thermo-Finnigan Delta XP^Plus^ Isotope Ratio Mass Spectrometer (IRMS). Carbon isotope δ^13^C (CO_2_) values are reported relative to the international reference standard Vienna Pee Dee Belemnite (VPDB). Precision of individual analyses of standards and samples is less than 0.1‰, itself calibrated relative to VPDB.

### UNA gas and carbon isotope analysis

Gas phase samples and water samples for dissolved gases were collected in pre-evacuated 250 ml Giggenbach bottles containing 50 ml of 4 N NaOH. Headspace gases (He, H_2_, O_2_, Ar, N_2_, CH_4_) were analyzed on an Agilent 7890a gas chromatograph (GC) equipped with two HP-molesieve columns (Agilent 19095P-MSO) held at 30 °C. Giggenbach bottle samples were connected to the GC via a vacuum line attached to a turbo pump. The headspace gas was introduced to the two parallel columns via two 250 µL loops through two 6-way valves switching simultaneously, in a stream of carrier gas. One side of the GC operated with Ar carrier gas whereas the other operated with H_2_ carrier gas, allowing the measurement of Ar on the side with H_2_ carrier gas and vice versa. Methane was analyzed on the H_2_ carrier gas column with a flame ionization detector. The other gases were analyzed on thermal conductivity detectors. The CH_4_ concentration in water samples (Online-only Table 1)^1^ was calculated from the mass of water collected in the Giggenbach bottle (by weighing the bottle before and after sampling) and the partial pressure of CH_4_ measured in the headspace, accounting for Henry´s Law partitioning of CH_4_ into the liquid portion of the sample. After GC analysis of the headspace gases the NaOH solution was extracted and measured for CO_2_ content by titration with 0.1N HCl. The CO_2_/CH_4_ ratio in gas samples (Online-only Table 1)^1^ is determined by calculating the total moles of CO_2_ and CH_4_ collected in the Giggenbach bottle. The error of the CH_4_ concentrations and CO_2_/CH_4_ ratios were estimated at less than 5% and less than 10%, respectively.

Carbon isotope compositions of gas samples from the 2017 campaign were analyzed at UNA on a Picarro G2201-I by acidification of NaOH solutions extracted from Giggenbach bottle samples. The concentration of dissolved CO_2_ in the solutions was previously determined by titration with 0.1 N HCl. Between 0.02 ml and 0.4 ml of sample solution was introduced into 15 ml septum vials which were loaded onto an Automate FX sample preparation device (autosampler). The autosampler introduced phosphoric acid to the vials to exsolve CO_2_, which was flushed from the vials with N_2_ and transferred to 0.5 L gas sampling bags in a Picarro Liaison Interface. The CO_2_ - N_2_ mixture was then transferred automatically to the Picarro G2201-I and analyzed in iCO_2_ low concentration N_2_ background mode for 8 minutes at a flow rate of ~25 ml/min. Carbon dioxide concentrations averaged around 2500 ppm, depending on the CO_2_ content of the NaOH sample solution and volume of sample loaded in the vial. δ^13^C_PDB_ values were calibrated against a set of 8 standards with values ranging from +2.42‰ to −37.21‰, including internationally accepted standards NBS19 and Carrara Marble. The linear calibration curve generated for the carbon isotope compositions by this method had an R^2^ of 0.99996 and slope of 1.00584. Reported δ^13^C_PDB_ values had uncertainties of <0.1‰ based on repeat analyses of standards and samples.

### Oxford noble gas isotope analysis

Noble gas analysis was also conducted in the Noble Laboratory at the University of Oxford (2017 samples), using a dual mass spectrometer setup, interfaced to a dedicated extraction and purification system^13^. Gases were collected in Cu-tubes, and then transferred to the extraction and purification line where reactive gases were removed by exposing gases to a titanium sponge held at 950 °C. The titanium sponge was cooled for 15 minutes to room temperature before gases were expanded to a dual hot (SAES GP-50) and cold (SAES NP-10) getter system, held at 250 °C and room temperature, respectively. A small aliquot of gases was segregated for preliminary analysis on a Hiden Analytical HAL-200 quadrupole mass spectrometer. All noble gases were then concentrated using a series of cryogenic traps; heavy noble gases (Ar-Kr-Xe) were frozen down at 15 K on an all stainless-steel finger and the He and Ne were frozen down at 19 K on a cold finger filled with charcoal. The temperature on the charcoal finger was then raised to 34 K to release only He, which was inlet into a Helix SFT mass spectrometer. Following He analysis, the temperature on the charcoal cryogenic trap was raised to 90 K to release Ne, which was inlet into an ARGUS VI mass spectrometer.

### Tokyo tech carbon isotope analysis

Water samples for carbon isotope analysis (2017 samples) of dissolved inorganic carbon (DIC) and dissolved organic carbon (DOC) were collected using a 50-mL syringes, filtered through a membrane syringe filters with a pore size of 0.22 µm (DISMIC–25AS; Advantec Toyo Kaisha, Tokyo, Japan), and directly injected into a pre-evacuated 50 mL serum bottle sealed with butyl rubber septa and an aluminum crimp. Water was subsampled (10 mL) for DIC measurement by nitrogen gas and transferred to a pre-evacuated 30 mL glass vial sealed with butyl rubber septum and an aluminum crimp. DIC concentrations and their δ^13^C values were measured using CO_2_ in the headspace of glass vials after a 1-h reaction with injected 0.5 mL H_3_PO_4_. DOC was also measured as CO_2_ in the headspace after the reaction of carbonate-free residue with 0.2 g sodium persulfate. The amount of CO_2_ and the isotopic values were measured using an Agilent 6890 N gas chromatograph attached to a Thermo-Finnigan Delta XP^Plus^. δ^13^C_PDB_ values were calibrated against five standards ranging from −27.57‰ to 2.52‰ including internationally accepted standards NBS18 and NBS19. The standard deviations were determined by 3 or more measurements.

The sediments surrounding the surface emanations of the springs were collected for total organic carbon (TOC) content and carbon isotopic compositions. The sediment samples were kept at 4 °C until transport to the laboratory. Glass vial samples were then stored at −80 °C until further treatment. First, the sediment samples were freeze-dried and then crushed into fine grains using a mortar. 50–100 mg of sediment samples were weighed and reacted with 1 M HCl solution until effervescence stopped, followed by a rinse with distilled water until the pH neutralized. The HCl treated sediment samples were dried and analyzed using an elemental analyzer (EA-1110; Thermo Fisher Scientific) coupled to a Thermo Fisher Scientific MAT 252 isotope ratio mass spectrometer (IRMS). Three laboratory standards (δ^13^C = −27.57‰, −19.60‰, and 2.52‰) were used for standardization, and the δ^13^C_PDB_ values have uncertainties of <0.2‰ based on repeat analyses of standards. The standard deviations were shown by repeated measurements of samples.

### University of tennessee cell counts

Samples for cell counts were taken in 2017 as close to the source spring as possible, usually in an outflow from a rock outcrop or a small surface pool that was rapidly being refilled by the source. Whole fluids (1 ml) were placed into a 2 ml plastic tube with a rubber o-ring screwcap (to prevent evaporation) containing 500 µl 3% paraformaldehyde solution in phosphate-buffered-saline (PBS) and kept at room temperature during return to the University of Tennessee, where they were weighed. Cell counts were determined on a Guava Easy Cyte 6HT-2L (Millipore) flow cytometer. Triplicate aliquots of each sample (200 μL) were stained with 5× SybrGreen prior to analysis. Gating strategy was optimized using stained, unstained, and filtered controls. Total cell counts, and average carbon content of subsurface microbes^14^, were used to estimate contributions of cell biomass by multiplying the average number of cells in fluid samples by the volume of hosting rocks (from the trench to the arc – assuming a log increase of the isotherms moving toward the arc) up to 2 km depths, and using an average rock porosity (to obtain possible fluid amounts)^15^, and found that they represented a significant carbon reservoir.

### UNIVPM aliphatic hydrocarbons and polycyclic aromatic hydrocarbons (PAH) analysis

Aliphatic hydrocarbons (C10-C40) and polycyclic aromatic hydrocarbons (PAHs) (Online-only Table 2)^1^ were analyzed at the Dipartimento di Scienze della Vita e dell’Ambiente (DISVA), Università Politecnica delle Marche (UNIVPM), Italy, using conventional procedures based on gas chromatography with a flame ionization detector (FID) and HPLC with diode array and fluorimetric detection^16^. Briefly, aliphatic hydrocarbons (C10-C40) were extracted with hexane:acetone (2:1) in a microwave (110 °C for 25 min, 800 Watt) (Mars CEM, CEM Corporation, Matthews NC). After centrifugation at 3.000 × g for 10 min, the supernatants were purified with solid-phase extraction (Phenomenex Strata-X, 500 mg × 6 mL plus Phenomenex Strata-FL, 1000 mg × 6 mL) and then concentrated using a SpeedVac (RC1009; grade n-hexane and analyzed with a PerkinElmer GC) equipped with an Elite-5 capillary column (30 mm × 0.32 mm ID × 0.25 µm-df) and a FID. For quantitative determination, the system was calibrated with an unsaturated pair n-alkane standard mixture according to ENISO 9377-3 (Fluka 68281). For the analysis of PAHs, sediment samples were extracted using 0.5 M potassium hydroxide in methanol with microwave at 55 °C for 20 min (800 Watt) (CEM, Mars System). After centrifugation at 3.000 × g for 10 min, the methanol extracts were concentrated using a SpeedVac and purified with solid-phase extraction (Octadecyl C18, 500 mg × 6 mL, Bakerbond). A final volume of 1 mL was recovered with pure, analytical HPLC gradient grade acetonitrile; HPLC analyses were carried out in a water-acetonitrile gradient by fluorimetric and diode array detection. The PAHs were identified according to the retention times of an appropriate pure standards solution (EPA 610 Polynuclear Aromatic Hydrocarbons Mix), and classified as low molecular weight (LMW: naphthalene, acenaphthylene, 1-methyl naphthalene, 2-methyl naphthalene, acenaphthene, fluorene, phenanthrene, anthracene) or high molecular weight (HMW: fluoranthene, pyrene, benzo(a)antrhacene, chrysene, 7,12-dimethyl-benzo(a)anthracene, benzo(b)fluoranthene, benzo(k)fluoranthene, benzo(a)pyrene, dibenzo(a,h)anthracene, benzo(g,h,i)perylene, indeno(1,2,3-cd)pyrene). Accuracy and precision were checked analyzing both pure standard solutions and reference materials (NIST 1944) and the obtained concentrations were always within the 95% confidence intervals of certified values. Aliquots of all the samples were dried in an oven at 60 °C for at least 8 h, up to obtain a constant weight, in order to quantify the interstitial water content, allowing to express all the analyzed chemicals as a function of the dry weight (d.w.) of the sediments.

## Data Records

All data records have been placed in the EarthChem repository (10.1594/IEDA/111271)^17^. For carbon isotope (δ^13^C_PDB_) analyses (DIC, DOC, TOC), three individual laboratory standards (δ^13^C = −27.57‰, −19.60‰, and 2.52‰) were used for standardization. Carbon isotope values in Online-only Table 1 have uncertainties of <0.2‰ (at the 95% confidence interval) based on repeat analyses of standards. The standard deviations were calculated by repeated measurements of those standards. δ^13^C_PDB_ values of CO_2_ (Online-only Table 1) were calibrated against a set of 8 standards with values ranging from +2.42‰ to −37.21‰, including internationally accepted standards NBS19 and Carrara Marble. The linear calibration curve generated for the carbon isotope compositions by this method had an R^2^ of 0.99996 and slope of 1.00584. Reported δ^13^C_PDB_ values of CO_2_ had uncertainties of <0.1‰ based on repeat analyses of standards and samples. For helium isotope (^3^He/^4^He) analyses, air (=1R_A_) was used for standardization. Helium isotope values in Online-only Table 1 have uncertainties of <5.1‰ (at the 95% confidence interval) based on repeat analyses of air standards. The standard deviations were calculated by repeated measurements of air standards. At Oxford, helium isotope (^3^He and ^4^He) values were measured simultaneously for 100 cycles, using an integration time of 4.19 seconds per cycle and regressed back to time zero to calculate a representative starting value at sample inlet. A similar approach was taken at SIO for He isotope analysis, however peak jumping between ^3^He and ^4^He was done. Cell count uncertainties in Online-only Table 2 are <10% and are reported as one standard deviation of the triplicate counts. PAH values in Online-only Table 2 were cross calibrated against both pure standard solutions and reference materials (NIST 1944) and the determined concentrations are all within the 95% confidence intervals of certified values. NA = Not Available due to insufficient gas (or material) to make the measurement.

## Technical Validation

Samples were collected over a nine-year span and analyzed for He and C isotopes in three different laboratories, therefore technical validation of reproducibility between labs is necessary. Notably, all samples were collected during the dry season in an effort to minimize seasonal effects (Online-only Table 1). Considering this, the data from the various labs are in good agreement. For example, fluids were collected at the Sabana Grande site in 2008, 2010 and again in 2017; C-isotope values of −12.69‰ (2017; Japan) agree well with −12.75‰ (2008; SIO), −12.79‰ (2008; SIO) and −13.43‰ (2012; SIO). He-isotope values for this site were 2.66 ± 0.13 R_A_ (2017; Oxford), 0.60 ± 0.03 R_A_ (2008; SIO) and 1.04 ± 0.11 R_A_ (2012: SIO). When considering the reproducibility of He isotope measurements, it is essential to consider the amount of air contamination in a given sample, which is estimated using the relative amount of He and Ne, expressed as the X-value (^4^He/^20^Ne normalized to air). At Sabana Grande, the most pristine sample (highest X-value) yielded the highest He isotope value of 2.66 R_A_. Helium isotopes were also measured in samples collected at the Pueblo Antiguo site in 2010 (SIO), 2012 (SIO) and 2017 (Oxford), and in the two samples with high X-values (>5), the He isotopes measured at different laboratories are within analytical error (Oxford, 2017 = 4.34 ± 0.22 R_A_ and SIO, 2010 = 4.51 ± 0.11 R_A_).

## Data Availability

There is no custom code used in the generation or processing of these datasets. The spreadsheets used for the calcite precipitation model and the carbon flux calculations are included in the original research paper^1^.

## Online-only Tables

**Online-only Table 1:** Sample collection information, as well as helium and carbon isotope data. X-values represent air-normalized ^4^He/^20^ Ne values (considering solubility in water for fluid samples^18^), which are used to determine air-corrected ^3^He/^4^He values (R_C_/R_A_) of the samples^19^. CO_2_/^3^He is calculated using raw He-isotope values (R/R_A_). These data were originally reported in the supplementary material of Barry *et al*., 2019^1^.

Station	Area	Site name	Distance trench [km]	Province	Date collected	Altitude [m]	Temp [°C]	pH	DIC	DIC δ13C	DOC	DOC δ^13^C	TOC	TOC δ13C	CO_2_	δ^13^C CO_2_ (gas)	phase	^3^He/^4^He	^3^He/^4^He	X-value	CO_2_/^3^He	CH_4_/CO_2_	CH_4_
					(MM.DD.YYYY)				[mmol C L^−1^]		[mmol C L^−1^]		[C w/w%]		[mmol mol^−1^]			(R/Ra)	(Rc/Ra)			(gas phase)	(water phase)
BR170218_1	Northern	Blue River Spring 1	174	Arc	02.18.2017	437	59	6.16	9.56 ± 0.7	0.23 ± 0.2	0.8 ± 0.0	−11.16 ± 2.0	1.65 ± NA	−25.75 ± NA	947	−3.66	G	6.39	6.52 ± 0.33	32.29	1.36E + 10	NA	NA
BR170218_2	Northern	Blue River Spring 2	174	Arc	02.18.2017	434	53.8	5.87	9.31 ± 1.0	0.96 ± 0.3	0.89 ± 0.1	−10.75 ± 2.0	1.24 ± 0.2	−28.31 ± 0.7	NA	NA	G	6.39	6.52 ± 0.33	32.29	1.36E + 10	1.81E-03	NA
BQ170218	Northern	Borinquen	159	Arc	02.18.2017	535	88.9	2.11	NA	NA	NA	NA	0.10 ± NA	−18.88 ± NA	937	−1.37	G	5.03	5.13 ± 0.26	33.24	1.01E + 11	NA	NA
ET170220_1	Northern	Eco Thermales	173	Arc	02.20.2017	368	40	6.06	NA	NA	NA	NA	1.05 ± 0.33	−28.68 ± 0.37	NA	NA	G	NA	NA	NA	NA	NA	NA
SI170217	Northern	El Sitio	100	Outer Forearc	02.17.2017	36	35.9	9.83	2.24 ± 0.5	−6.43 ± 0.6	0.4 ± 0.0	−18.27 ± 2.8	4.45 ± 0.6	−27.49 ± 0.2	67	NA	G	0.49	0.39 ± 0.02	5.05	2.17E + 09	NA	NA
TC170221	Central	El Tucano	173	Arc/Backarc	02.21.2017	553	60	6.24	13.57 ± 1.1	−2.36 ± 0.7	1.07 ± 0.1	−7.94 ± 1.9	5.04 ± 6.1	−28.17 ± 0.6	965	−4.54	G	6.51	6.57 ± 0.33	76.54	2.40E + 10	9.75E-04	NA
EP170215	Central	Espabel	74	Outer Forearc	02.15.2017	NA	26.4	9.99	0.55 ± 0.1	−21.57 ± 1.8	0.22 ± 0.0	−25.48 ± 3.6	3.1 ± 4.0	−27.89 ± 0.8	0	NA	W	NA	NA	NA	NA	NA	4.05E-03
ES170215_1	Central	Estrada	74	Outer Forearc	02.15.2017	122	27.9	9.75	NA	NA	NA	NA	NA	NA	NA	NA	W	NA	NA	NA	NA	NA	NA
FA170219_1	Northern	Finca Ande	140	Forearc/Arc	02.19.2017	109	55.2	5.93	14.22 ± 1.0	2.71 ± 0.3	0.98 ± 0.1	−6.64 ± 1.7	1.23 ± NA	−26.46 ± NA	995	−1.36	G	4.17	4.36 ± 0.22	14.14	8.98E + 11	1.86E-05	NA
HN170219	Northern	Las Hornillas	167	Arc	02.19.2017	765	87.9	1.82	1.12 ± 0.1	−2.84 ± 0.3	0.67 ± 0.1	−16.05 ± 2.5	1.01 ± 0.0	−16.75 ± 0.4	981	−2.29	G	6.68	6.73 ± 0.34	87.55	1.18E + 11	2.63E-03	NA
MT170219	Northern	Mouse Trap	153	Forearc/Arc	02.19.2017	166	59.1	6.32	21.7 ± 1.1	3.55 ± 0.3	1.66 ± 0.1	−7.38 ± 1.7	0.25 ± NA	−24.05 ± NA	988	−0.84	G	3.2	3.57 ± 0.18	5.58	6.85E + 11	7.49E-04	NA
PU170224	Central	Poas Volcano fumarole	165	Arc	02.24.2018	2335	90	NA	NA	NA	NA	NA	0.03 ± NA	−20.69 ± NA	901.3	−4.2	G	6.5	6.48 ± 0.32	244	1.51E + 10	NA	NA
PL170224	Central	Poas Volcano lake	165	Arc	02.24.2018	2334	37.6	0.85	0.86 ± 0.1	−2.97 ± 1.0	0.57 ± 0.1	−20.7 ± 3.01	0.02 ± NA	−22.13 ± NA	971.2	−4	W	NA	NA	NA	NA	NA	NA
PF170222	Central	Pompilo’s finca	202	Backarc	02.22.2017	53	28.7	5.81	23.72 ± 4.0	1.24 ± 0.4	1.16 ± 0.4	−5.15 ± 1.6	1.66 ± 0.1	−25.14 ± 0.3	998	−3.64	G	1.27	1.72 ± 0.09	1.3	3.74E + 13	1.78E-05	NA
PA170214	Northern	Pueblo Antiguo	142	Forearc	02.14.2017	262	45	7.62	NA	NA	NA	NA	NA	NA	36	NA	G	4.29	4.34 ± 0.22	48.27	1.88E + 08	NA	NA
QN170220	Northern	Quebrada Naranja	174	Arc	02.20.2017	429	22.9	5.9	2.81 ± 0.2	−1.95 ± 0.4	0.42 ± 0.1	−13.82 ± 2.7	0.20 ± NA	−24.64 ± NA	926	−2.76	G	3.15	4.89 ± 0.24	1.82	4.07E + 10	6.98E-02	NA
RS170216	Northern	Ranchero El Salitral	99	Outer Forearc	02.16.2017	82	29.4	9.96	0.25 ± 0.0	−16.04 ± 1.4	0.3 ± 0.1	−24.55 ± 3.5	1.19 ± 0.3	−27.64 ± 0.8	NA	NA	W	2.64	2.66 ± 0.13	30.67	1.18E + 09	NA	8.45E-04
RV170221	Central	Recreo Verde	176	Arc/Backarc	02.21.2017	557	42.7	6.19	19.73 ± 3.2	−0.28 ± 0.7	2.87 ± 0.3	−4.71 ± 1.6	NA	NA	994	−4.8	G	5.63	6.68 ± 0.33	4.38	2.19E + 11	2.74E-06	NA
CY170214	Central	Rio Cayuco	141	Forearc	02.14.2017	184	72	6.31	5.24 ± 0.8	−4.99 ± 0.5	1.82 ± 0.2	−7.78 ± 1.7	0.25 ± 0.1	−23.21 ± 0.3	995	−2.4	G	0.86	0.59 ± 0.03	1.24	1.81E + 11	1.80E-06	NA
SR170216	Northern	Salitral el Rincon	90	Outer Forearc	02.16.2017	66	33	9.06	NA	NA	NA	NA	NA	NA	0	NA	G	NA	NA	NA	NA	NA	NA
SL170214	Central	Santa Lucia	140	Forearc	02.14.2017	165	57	6.12	5.69 ± 0.3	−5.28 ± 0.5	6.3 ± 1.2	−0.65 ± 1.4	3.15 ± 1.1	−28.56 ± 0.2	NA	NA	W	3.77	3.84 ± 0.19	34.7	1.01E + 11	NA	NA
ST170223	Central	Santa Theresa	164	Arc	02.23.2017	2209	55.8	4.51	9.94 ± 1.5	−3.88 ± 0.5	0.8 ± .0.1	−11.40 ± 2.1	NA	NA	187.3	−4.34	W	NA	NA	NA	NA	NA	NA
VC170218	Northern	Volcancito	174	Arc	02.18.2017	436	59.8	5	6.13 ± 0.3	−1.28 ± 0.2	1.0 ± 0.1	−10.22 ± 1.9	0.33 ± NA	−18.24 ± NA	925	−4.17	G	6.79	6.88 ± 0.34	50.47	5.11E + 09	2.19E-03	NA

**Online-only Table 2:** Sample location information, cell counts and polycyclic aromatic hydrocarbon (PAH) data. These data were originally reported in the supplementary material of Barry et al., 2019^1^.

Station	Site name	Latitude [°N]	Longitude [°E]	×10^6^ cells mL^−1^ fluids	Low MW PAHs [ng g^−1^ dry sediment]	High MW PAHs [ng g^−1^ dry sediment]	Total PAHs [ng g^−1^ dry sediment]	Total AH [µg g^−1^ dry sediment]
BR170218_1	Blue River Spring 1	10.89837	−85.32839	1.10 ± 0.039	60.3	16	76.3	146.8
BR170218_2	Blue River Spring 2	10.89837	−85.32853	1.20 ± 0.081	8.2	5.6	13.8	168.7
BQ170218	Borinquen	10.81088	−85.41371	NA	10.7	4.7	15.4	118
CR08-12	Colorado de Abangares	10.18847	−85.10717	NA	NA	NA	NA	NA
CR08-57	Colorado de Abangares	10.18847	−85.10717	NA	NA	NA	NA	NA
ET170220_1	Eco Thermales	10.48401	−84.67585	0.05 ± 0.03	18.6	0.9	19.5	77
SI170217	El Sitio	10.30124	−85.61055	0.018 ± 0.01	13.8	9	22.8	92
CR08-10	El Sitio	10.30124	−85.61061	NA	NA	NA	NA	NA
TC170221	El Tucano	10.36649	−84.38121	0.052 ± 0.03	94.8	71.7	166.5	133.5
EP170215	Espabel	9.901885	−85.45433	NA	9.9	4.3	14.2	48.1
ES170215_1	Estrada	9.899005	−85.45351	0.028 ± 0.07	NA	NA	NA	NA
FA170219_1	Finca Ande	10.33684	−85.0695	1.31 ± 0.13	23.5	13.2	36.6	274
HN170219	Las Hornillas	10.71282	−85.1774	NA	30.2	19.5	49.7	62.1
MT170219	Mouse Trap	10.59577	−85.23845	0.07 ± 0.01	10.9	15.6	26.5	31.3
CR08-09	Paraiso Town	10.18403	−85.79533	NA	NA	NA	NA	NA
PU170224	Poas Volcano fumarole	10.19678	−84.22989	NA	12.1	3.1	15.3	37.1
PL170224	Poas Volcano lake	10.19678	−84.22989	0.003 ± 0.002	6.4	3	9.3	46.1
PF170222	Pompilo’s finca	10.51847	−84.11518	1.00 ± 0.29	56.8	5.5	62.4	225.8
PA170214	Pueblo Antiguo	10.28311	−84.9293	NA	NA	NA	NA	NA
CR10-13	Pueblo Antiguo	10.28319	−84.92919	NA	NA	NA	NA	NA
CR12-36	Pueblo Antiguo	10.28328	−84.92925	NA	NA	NA	NA	NA
QN170220	Quebrada Naranja	10.49557	−84.69671	0.18 ± 0.04	20.6	8.9	29.5	113
RS170216	Ranchero El Salitral	10.23233	−85.5316	0.36 ± 0.06	330	209	539	94.7
CR08-02	Ranchero El Salitral	10.23211	−85.53153	NA	NA	NA	NA	NA
CR12-29	Ranchero El Salitral	10.23231	−85.53153	NA	NA	NA	NA	NA
RV170221	Recreo Verde	10.32158	−84.24369	1.37 ± 7.2	NA	NA	NA	NA
CY170214	Rio Cayuco	10.2875	−84.95552	NA	6.5	5.1	11.6	64.4
SM170216	Sabana Grande	10.17741	−85.47971	0.035 ± 0.023	NA	NA	NA	NA
CR08-08	Sabana Grande	10.17906	−85.48014	NA	NA	NA	NA	NA
CR08-21	Sabana Grande	10.17906	−85.48014	NA	NA	NA	NA	NA
CR12-14	Sabana Grande	10.17869	−85.47958	NA	NA	NA	NA	NA
CR08-03	Salitrado San Martin	10.15983	−85.45964	NA	NA	NA	NA	NA
CR08-14	Salitrado San Martin	10.15983	−85.45964	NA	NA	NA	NA	NA
CR12-89	Salitrado San Martin	10.15983	−85.45964	NA	NA	NA	NA	NA
SR170216	Salitral el Rincon	10.25371	−85.68312	1.07 ± 0.16	NA	NA	NA	NA
CR08-01	Salitral Vigia	10.10683	−85.28606	NA	NA	NA	NA	NA
CR10-08*	Santa Lucia	10.2875	−84.9555	NA	NA	NA	NA	NA
CR10-10*	Santa Lucia	10.2875	−84.9555	NA	NA	NA	NA	NA
SL170214	Santa Lucia	10.2906	−84.97244	NA	89.5	1,565.60	1,655.10	78.7
ST170223	Santa Theresa	10.00294	−83.82751	0.64 ± 0.13	NA	NA	NA	NA
VC170218	Volcancito	10.89785	−85.32646	2.32 ± 0.37	23.1	2.8	25.9	181
